# Secondary neoplasms in survivors of pediatric acute lymphoblastic leukemia and lymphoblastic lymphoma: a single-center, retrospective study

**DOI:** 10.3389/fped.2025.1530832

**Published:** 2025-01-28

**Authors:** Eri Ushida, Hidemi Toyoda, Atsushi Kohso, Yosuke Okumura, Kaori Niwa, Takahiro Ito, Mari Morimoto, Ryo Hanaki, Keishiro Amano, Shotaro Iwamoto, Takao Deguchi, Hiroki Hori, Masahiro Hirayama

**Affiliations:** ^1^ Department of Pediatrics, Mie University Graduate School of Medicine, Tsu, Japan; ^2^ Division of Cancer Immunodiagnostics, Children’s Cancer Center, National Center for Child Health and Development, Tokyo, Japan; ^3^ Department of Medical Technology Course, Suzuka University of Medical Science, Suzuka, Japan

**Keywords:** acute lymphoblastic leukemia, lymphoblastic lymphoma, secondary neoplasm, cranial radiotherapy, total body irradiation

## Abstract

**Background:**

Acute lymphoblastic leukemia (ALL)-based therapeutic regimens have markedly improved the survival of children with ALL and lymphoblastic lymphoma (LBL). However, survivors are at risk of secondary neoplasms. Few studies on such secondary neoplasms have been conducted outside of Europe and the United States. The aim of this study was to evaluate the incidence of, risk factors for, and outcomes of secondary neoplasms in long-term survivors of ALL and LBL at a tertiary pediatric oncology center in Mie prefecture, Japan.

**Procedure:**

We retrospectively reviewed 188 patients with ALL and LBL who were treated with an ALL-based therapeutic regimen at Mie University Hospital from January 1, 1977 to December 31, 2022 and followed up.

**Results:**

Ten patients developed secondary neoplasms, with 10-year and 20-year cumulative incidences of 2.9% [standard error (SE) = 1.5%] and 5.5% (SE = 2.3%), respectively. The median interval between the primary-cancer diagnosis and secondary-neoplasm diagnosis was 18.5 years (range: 7.8–41.7 years). All 10 secondary neoplasms were central nervous system (CNS) tumors (6 meningiomas and 4 high-grade gliomas). Radiotherapy (*p* = 0.007) and CNS involvement in the primary cancer (*p* < 0.001) increased the risk of secondary neoplasms among long-term survivors. Gliomas occurred significantly earlier than meningiomas (*p* = 0.047), and three patients died of secondary neoplasms (all gliomas).

**Conclusions:**

As secondary gliomas occurred earlier than meningiomas and are associated with poor outcomes, physicians should take great pains to minimize their risk to improve long-term survival and quality of life.

## 1 Introduction

Acute lymphoblastic leukemia (ALL) is the most common childhood cancer, accounting for approximately 20%–30% of pediatric cancers (1). The 5-year survival of children with ALL improved from <35% in the early 1970s to 90% in 2010 (2, 3). Lymphoblastic lymphoma (LBL), the second most common type of non-Hodgkin lymphoma in children, is usually treated with an ALL-based therapeutic regimen because ALL and LBL are on the same disease spectrum (4, 5). The event-free and overall survival rates of children with LBL now exceed 80% (6, 7). The number of long-term survivors of childhood ALL and LBL has risen; however, many survivors experience premature mortality and excess morbidity in the form of secondary neoplasms, chronic health conditions, neurocognitive impairment, and a self-reported poor health status because of cancer treatment (2).

Secondary neoplasms are considered the most fatal and life-threatening late effects among long-term survivors of childhood ALL and LBL, and several studies have reported the incidence of, type of, risk factors for, and outcomes of secondary neoplasms (8–12). Reported risk factors for secondary-neoplasm development are cancer-predisposing conditions, cranial radiotherapy (CRT), the treatment regimen, female sex, younger age at primary cancer diagnosis, initial characteristics of the primary cancer, radiation dose, and allogeneic hematopoietic stem-cell transplantation (allo-HCT) (8, 10, 11, 13–17). Interestingly, Moser et al. reported that an ALL-based therapeutic regimen was a significant risk factor for secondary neoplasms in long-term survivors of LBL (10).

ALL-based therapeutic regimens consist of a four-drug induction, central nervous system (CNS) prophylaxis, consolidation therapy, and maintenance therapy for up to 2 years (18). Although recent ALL-based, intrathecal, systemic chemotherapeutic regimens have eliminated prophylactic CRT without jeopardizing leukemia control in the CNS (19), CRT is still administered as a therapeutic approach for ALL and LBL with CNS involvement. CRT may also be administered as a part of total body irradiation (TBI) within the conditioning regimen of children selected to undergo allo-HCT.

The observation that an ALL-based therapeutic regimen is a risk factor for secondary neoplasms in LBL prompted us to analyze the development of secondary neoplasms in patients who received ALL-based therapeutic regimens in our hospital. Furthermore, the majority of studies on secondary neoplasms have been conducted in Europe and the United States, and the cumulative incidence of secondary neoplasms has varied from less than 1% to 10% or more (8–13, 15, 16). Therefore, we aimed to retrospectively evaluate the cumulative incidence, clinical features, and outcomes of secondary neoplasms in long-term survivors of ALL and LBL who were treated with an ALL-based therapeutic regimen and followed up at our hospital in Japan.

## 2 Methods

### 2.1 Patients

Mie University Hospital is a tertiary referral center for pediatric oncology in Mie Prefecture, Japan. Patients with primary ALL or primary LBL treated with an ALL-based therapeutic regimen from January 1, 1977 to December 31, 2022 and still being followed up at Mie University Hospital were eligible for the study. Study participants were selected from among these survivors according to the following inclusion criteria: (a) age ≤18 years at the time of primary cancer diagnosis, (b) ≥1 year of follow-up at Mie University Hospital after the end of treatment, and (c) availability of outcome information. Patients lost to follow-up were excluded from the study.

The study protocol was approved by the institutional ethics committee (identifier: H2023-228 and H2020-129). The study was performed in accordance with the principles of the Declaration of Helsinki.

### 2.2 Follow-up and data collection

We obtained detailed information on sex, age at diagnosis of the primary cancers, and treatment for primary cancers, including cumulative radiation doses and cumulative doses of anticancer agents. For alkylating agents, the conversion factor for the cyclophosphamide-equivalent dose was 1:4 (i.e., 1 mg cyclophosphamide equals 4 mg ifosfamide). For anthracyclines, the conversion factors for the doxorubicin-equivalent dose were 1:0.5 (i.e., 1 mg doxorubicin equals 0.5 mg daunorubicin) and 1:10 (i.e., 1 mg doxorubicin equals 10 mg mitoxantrone). Patients were followed up at least annually in the outpatient clinic at our hospital. A secondary neoplasm was defined as a histologically or radiologically distinct neoplasm that developed after diagnosis of the primary cancer, for which it was possible to rule out metastasis or recurrence of the primary cancer. We collected the following data regarding secondary-neoplasm diagnosis: date of diagnosis; histological or radiological reports, including cytogenetic findings; site of secondary neoplasm; interval to secondary neoplasm; and outcomes. The time at risk for a secondary neoplasm was computed from the date of primary-cancer diagnosis to the date of secondary-neoplasm diagnosis, death, or last contact, whichever came first. The end of the follow-up period was December 31, 2023.

### 2.3 Statistical analysis

The cumulative incidence of secondary neoplasms over time was calculated using competing-risk methods, with death regarded as a competing event. In the regression model, we evaluated sex, age at primary-cancer diagnosis, CNS involvement, allo-HCT, radiotherapy, and chemotherapy. Age at primary-cancer diagnosis was analyzed as a categorical variable according to quartiles (<5 years, ≥5 years and <10 years, ≥10 years and <15 years, and ≥15 years). Primary ALL and LBL were classified into three (precursor B-ALL, T-ALL, and Philadelphia-positive ALL) and two (T-LBL and B-LBL) categories, respectively. Survival analysis was conducted using the Kaplan–Meier method (log-rank method for comparison). Data were analyzed using the BellCurve for Excel software (Social Survey Research Information Co., Ltd. Tokyo, Japan). Statistical significance was set at *p* < 0.05.

## 3 Results

### 3.1 Patient characteristics

A total of 188 survivors participated in the study and their characteristics are summarized in Table 1. Of 188 patients, 171 were diagnosed with ALL and 17 with LBL. The median age at primary-cancer diagnosis was 5.5 (range, 0.2–18.5) years. The median duration of follow-up from the end of treatment for primary cancer was 14.6 (range, 1.0–43.8; mean, 15.5) years. The duration of follow-up was <5 years for 37 patients, ≥5 but <10 years for 31 patients, ≥10 but <15 years for 30 patients, ≥15 but <20 years for 30 patients, and ≥20 years for 60 patients. Twenty-seven patients underwent allo-HCT, and 63 received radiotherapy, either CRT or TBI.

**Table 1:** Characteristics and distribution of childhood cancer survivors included in this study.

	All patients		Patients with secondary neoplasms		20 years’ cumulative incidence	*p*
	*N*	(%)	*N*	(%)	% (SE)	
**Total**	**188**		**10**		**5.5** **(2.3)**	
Sex						
Male	101	(53.7)	4	(40.0)	2.9 (2.0)	0.40
Female	87	(46.3)	6	(60.0)	7.9 (4.0)	
Age at diagnosis of primary cancer						
<5 years	91	(48.4)	5	(50.0)	3.3 (2.3)	0.75
≥5 years and <10 years	53	(28.2)	2	(20.0)	3.0 (3.0)	
≥10 years and <15 years	38	(20.2)	3	(30.0)	14.5 (8.5)	
≥15 years	6	(3.2)	0	(0)	0	
Calender year of treatment						
1977–1979	2	(1.0)	0	(0)		
1980–1989	18	(9.6)	4	(40.0)		
1990–1999	38	(20.2)	3	(30.0)		
2000–2009	53	(28.2)	1	(10.0)		
2010–2019	59	(31.4)	2	(20.0)		
2020–2022	18	(9.6)	0	(0)		
Primary neoplasms						
ALL	171	(91.0)	8	(80.0)	4.6 (2.4)	0.45
B precursor ALL (Ph-)	155	(82.4)	7	(70.0)		
T-ALL	11	(5.9)	1	(10.0)		
Ph + ALL	5	(2.7)	0	(0)		
LBL	17	(9.0)	2	(20.0)	14.4 (9.5)	
T-LBL	15	(8.0)	2	(20.0)		
B-LBL	2	(1.1)	0	(0)		
CNS involvement						
Yes	7	(3.7)	2	(20.0)	25.0 (21.7)	<0.001
No	181	(96.3)	8	(80.0)	4.9 (2.3)	
allo-HCT						
Yes	27	(14.4)	2	(20.0)	4.6 (2.4)	0.48
No	161	(85.6)	8	(80.0)	11.8 (7.9)	
Radiotherapy						
Yes	63	(33.5)	10	(100.0)	12.0 (4.6)	0.0069
No	125	(66.5)	0	(0)	0	

ALL, acute lymphoblastic leukemia; T, T-cell; LBL, lymphoblastic lymphoma; Ph, Philadelphia chromosome; B, B-cell; CNS, central nervous system; allo-HCT, allogenieic hematopoietic stem-cell transplantation; SE, standard error.

**Figure 1:**
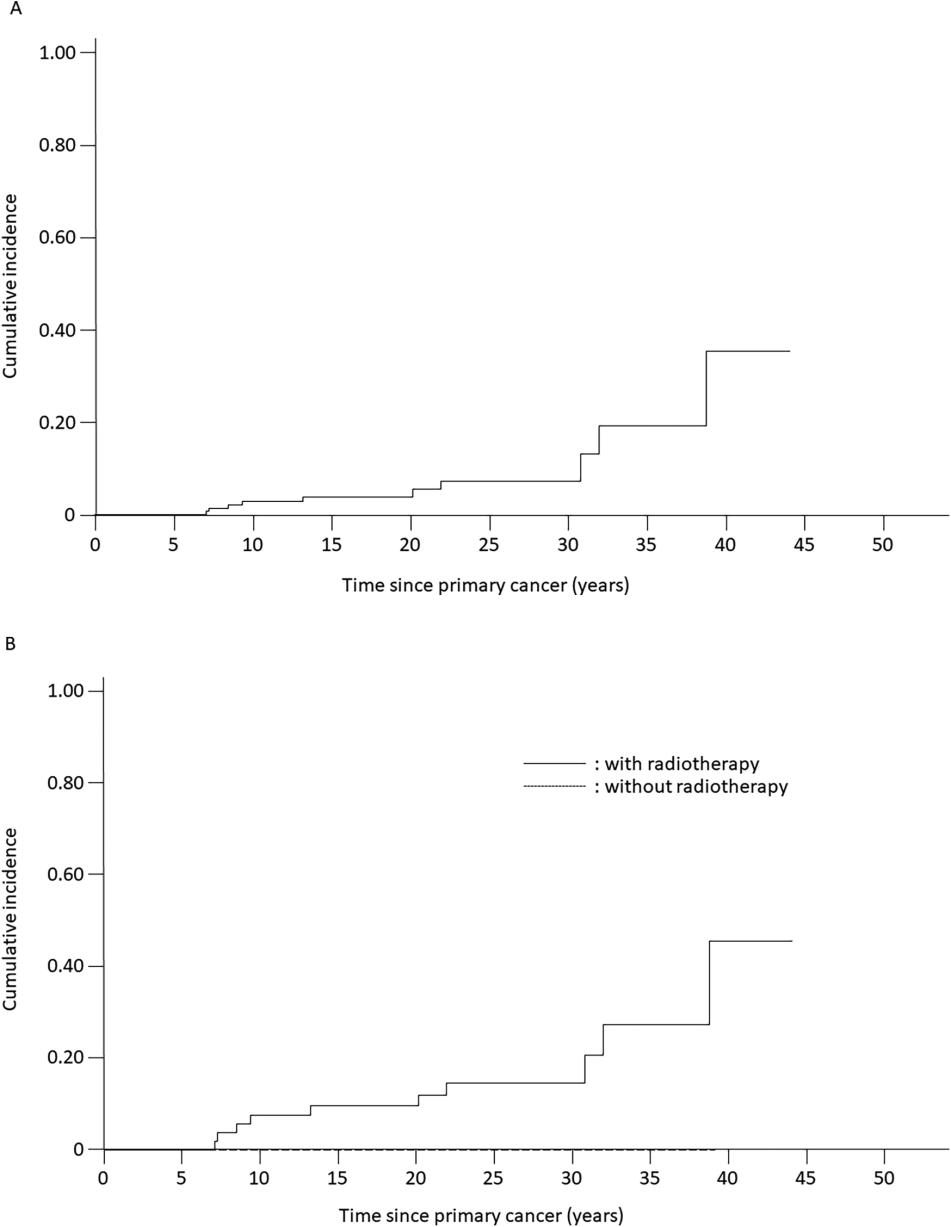
Estimated cumulative incidence of secondary neoplasms among patients with acute lymphoblastic leukemia (ALL) or lymphoblastic lymphoma who were treated with ALL-based therapeutic regimens. **(A)** Cumulative incidence of secondary neoplasms for all analyzed patients (5.5% ± 2.3%). **(B)** Cumulative incidence of secondary neoplasms for patients treated with radiotherapy (12.0% ± 4.6%) compared with that of patients treated without radiotherapy (0%). Solid line, patients receiving radiotherapy; dotted line, patients not receiving radiotherapy.

Of the 188 patients, 10 developed secondary neoplasms (Table 1). The cumulative incidence of secondary neoplasms at 10 and 20 years was 2.9% [standard error (SE) = 1.5%] and 5.5% (SE = 2.3%), respectively (Figure 1A). The primary-cancer diagnoses for the 10 patients with secondary neoplasms were B-cell precursor ALL (*n* = 7), T-cell LBL (*n* = 2), and T-cell ALL (*n* = 1). The median age at diagnosis of primary cancer was 4.5 (range, 2.9–14.3) years for patients who subsequently developed a secondary neoplasm, and the median age at diagnosis of the secondary neoplasm was 27.1 (range, 12.2–46.1) years (Table 2). The median time from primary-cancer diagnosis to secondary-neoplasm diagnosis was 18.5 (range, 7.8–41.7 years) years. No patient was diagnosed with secondary neoplasms within 5 years of the primary-cancer diagnosis. The analyzed group did not include patients with known cancer-predisposing conditions.

**Table 2:** Clinical characteristics of patients with secondary neoplasms.

	Diagnosis of primary malignancy	Age at onset	XRT	Latency between XRT and SN (years)	Symptoms	Diagnosis of SN	Age at SN	Treatment	Duration after SN (years)	Outcome
1	T-ALL	11.6	TBI, 12 Gy	7.0	Convulsion	High-grade glioma	19.4	OP, Chemo, XRT	2.3	Alive
2	T-LBL	2.9	CSI, 18 Gy	8.3	Convulsion	Meningioma	12.2	OP, XRT	10.3	Alive
3	BCP-ALL	9.9	TBI, 12 Gy	9.4	Right hemiplegia	High-grade glioma	19.3	OP, Chemo, XRT	3.1	DOD
4	T-LBL	14.3	CRT, 24 Gy	12.1	Convulsion	High-grade glioma	26.6	OP, Chemo, XRT	6.0	DOD
5	BCP-ALL	4.3	CRT, 15 Gy	14.0	Convulsion	High-grade glioma	19.3	Chemo, XRT	1.9	DOD
6	BCP-ALL	13.1	CRT, 18 Gy	20.8	Discomfort in eye	Meningioma	35.1	OP	3.2	Alive
7	BCP-ALL	4.1	CRT, 15 Gy	22.3	None	Meningioma	27.6	OP	5.0	Alive
8	BCP-ALL	3.3	CRT, 24 Gy	30.6	None	Meningioma	36.0	None	5.3	Alive
9	BCP-ALL	5.0	CRT, 24 Gy	34.8	None	Meningioma	40.0	None	7.0	Alive
10	BCP-ALL	4.5	CRT, 24 Gy	41.5	None	Meningioma	46.1	None	1.2	Alive

T, T-cell; ALL, acute lymphoblastic leukemia; LBL, lymphoblastic lymphoma; BCP, B-cell precursor; XRT, radiotherapy; TBI, total-body irradiation; CSI, craniospinal irradiation; CRT, cranial radiotherapy; SN, secondary neoplasm; OP, operation; Chemo, chemotherapy; DOD, died of the disease.

### 3.2 Risk factors for secondary neoplasms

**Figure 2:**
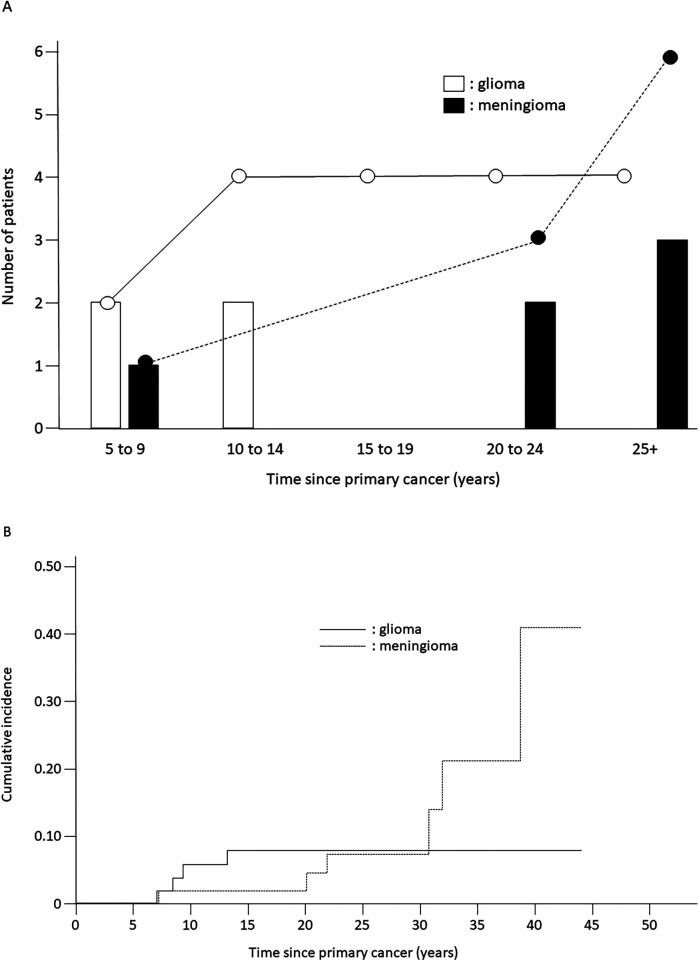
Incidence of secondary neoplasms. **(A)** Time to occurrence of subsequent glioma or meningioma from primary-cancer diagnosis in patients treated with acute lymphoblastic leukemia-based therapeutic regimens; white bars, white circles, and solid line: gliomas; black bars, black circles, and dotted line: meningiomas. **(B)** Cumulative incidence of secondary gliomas and meningiomas. Solid line: gliomas; dotted line: meningiomas.

The cumulative incidence of secondary neoplasms at 20 years was 14.4% (SE = 9.5%) among patients with LBL and 4.6% (SE = 2.4%) among patients with ALL (*p* = 0.45) (Table 1). Secondary neoplasms had a higher cumulative incidence at 20 years among patients with CNS involvement than those among patients without CNS involvement (25.0%, SE = 21.7% vs. 4.9%, SE = 2.3%, *p* < 0.001). No statistically significant differences in the cumulative incidence of secondary neoplasms were observed with respect to sex, age at primary diagnosis, or allo-HCT status. However, the cumulative incidence of secondary neoplasms was significantly higher among patients treated with radiotherapy (12.0% at 20 years, SE = 4.6%) than that among patients treated without radiotherapy (0% at 20 years, *p* = 0.007) (Table 1, Figure 1B). Interestingly, all secondary neoplasms were CNS tumors, either meningiomas (*n* = 6; World Health Organization grade I) or high-grade gliomas (*n* = 4; World Health Organization grade III) (Table 2). The median time to the occurrence of secondary neoplasms from the primary-cancer diagnosis was 11.1 (range, 7.8–15.1) years for gliomas and 28.0 (range, 9.2–41.7) years for meningiomas. Secondary gliomas occurred 7.0–14.0 years after radiotherapy, and three of the four patients were diagnosed before the age of 20 years (Table 2, Figures 2A,B). Conversely, secondary meningiomas tended to appear later, with five of six cases diagnosed ≥20 years after radiotherapy, and the numbers of secondary-meningioma diagnoses did not seem to decrease thereafter. The median radiation dose was 18.0 (range, 12.0–24.0) Gy among patients who developed secondary neoplasms, 13.5 (range, 12.0–24.0) Gy for gliomas and 21.0 (range, 15.0–24.0) Gy for meningiomas. No radiation dose–response relationship was observed in our study. Of the 10 patients with secondary CNS neoplasms, six were evaluated using brain magnetic resonance imaging owing to acute or chronic symptoms (convulsions, hemiplegia, or slight discomfort in the eyes). The four asymptomatic patients were discovered to have secondary CNS neoplasms, all meningiomas, upon routine brain magnetic resonance imaging performed because of a history of radiotherapy.

In addition to radiotherapy, exposure to ALL-based therapeutic regimens, including anticancer agents, may result in secondary neoplasms. Therefore, we examined whether exposure to anticancer agents increases the risk of secondary neoplasms owing to radiotherapy. Higher cumulative doses of anticancer agents were not associated with a higher incidence of secondary neoplasms among patients who received radiotherapy (Table 3).

**Table 3:** Risk factors for the development of secondary neoplasms after radiotherapy in long-term survivors with ALL or LBL.

	Patients with SNs		Patients without SNs		20 years’ cumulative incidence	*P*
	*n*	(%)	*n*	(%)	% (SE)	
**Total**	**10**	(100)	53	(100)	**5.5** **(2.3)**	
Sex						
Male	4	(40.0)	30	(56.6)	7.7 (5.3)	0.74
Female	6	(60.0)	23	(43.4)	15.3 (7.1)	
Age at diagnosis of primary malignancy						
<5 years	5	(50.0)	19	(35.8)	9.6 (6.5)	0.90
≥5 years and <10 years	2	(20.0)	16	(30.2)	9.1 (8.7)	
≥10 years and <15 years	3	(30.0)	16	(30.2)	19.3 (10.3)	
≥15 years	0	(0)	2	(3.8)	0	
Primary neoplasms						
ALL	8	(80.0)	47	(88.7)	9.5 (4.6)	0.30
B-cell precursor ALL (Ph-)	7	(70.0)	40	(75.5)		
T-ALL	1	(10.0)	3	(5.7)		
Ph + ALL	0	(0)	4	(7.5)		
LBL	2	(20.0)	6	(11.3)	28.6 (17.1)	
T-LBL	2	(20.0)	5	(9.4)		
B-LBL	0	(0)	1	(1.9)		
CNS status						
Positive	2	(20.0)	5	(9.4)	25.0 (21.7)	0.02
Negative	8	(80.0)	48	(90.6)	10.8 (4.6)	
Radiotherapy						
CRT (<15 Gy)	2	(20.0)	7	(13.2)	16.7 (15.2)	0.50
CRT (18 Gy)	2	(20.0)	19	(35.8)	10.4 (7.0)	
CRT (≥20 Gy)	4	(40.0)	10	(18.9)	6.7 (6.4)	
TBI (12 Gy)	2	(20.0)	17	(32.1)	20.5 (13.1)	
Anthracyclines^a^^,^^c^						
≤200 mg/m^2^ BSA	6	(66.7)	25	(48.1)	20.3 (8.3)	0.11
>200 mg/m^2^ BSA	3	(33.3)	27	(51.9)	4.4 (4.3)	
Alkylating agents^b^^,^^c^						
CPM ≤2 g/m^2^ BSA	3	(33.3)	2	(3.8)	16.7 (15.2)	0.44
CPM >2 g/m^2^ BSA	6	(66.7)	50	(96.2)	15.6 (5.9)	
Etoposide^c^						
≤800 mg/m^2^ BSA	8	(88.9)	36	(69.2)	13.3 (5.6)	0.74
>800 mg/m^2^ BSA	1	(11.1)	16	(30.8)	8.3 (8.0)	

Cumulative drug doses were calculated according to the actual dose administered to patients.

ALL, acute lymphoblastic leukemia; LBL, lymphoblastic lymphoma; Ph, Philadelphia chromosome; T, T-cell; B, B-cell; CNS, central nervous system; TBI, total-body irradiation; CRT, cranial radiotherapy; BSA, body surface area; CPM, cyclophosphamide; SN; secondary neoplasm; SE, standard error.

^a^ Doxorubicin-equivalent dose was calculated at a 1:1 ratio for doxorubicin and daunorubicin.

^b^ The conversion factor for the cyclophosphamide equivalent dose was 1:4 (i.e., 1 mg cyclophosphamide equals 4 mg ifosfamide).

^c^ Information about the cumulative dose of anticancer agents was missing for two patients.

### 3.3 Outcome after secondary neoplasms

**Figure 3:**
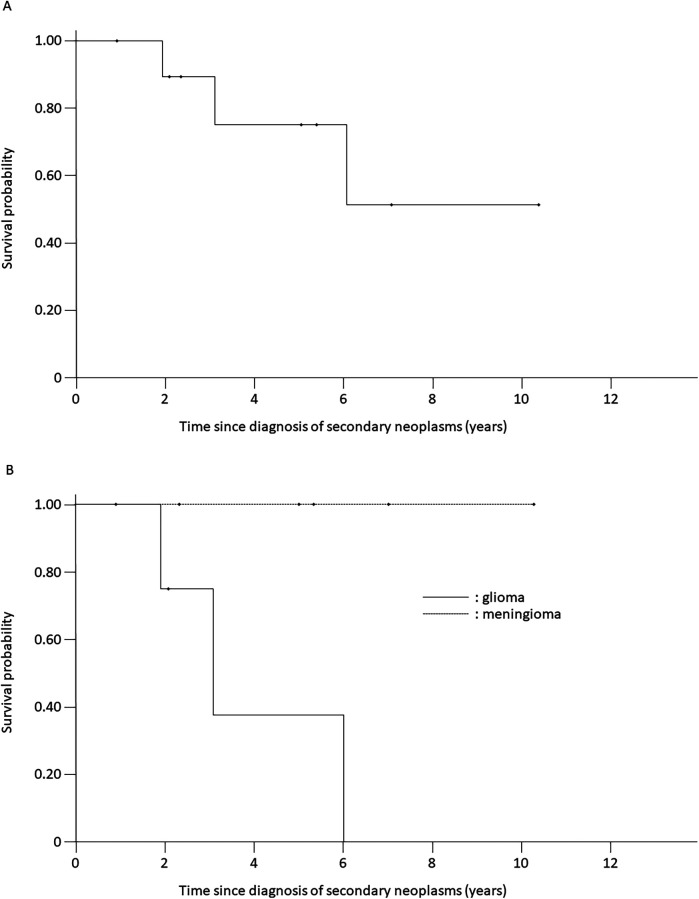
Kaplan–meier survival curves of patients with secondary neoplasms. **(A)** Overall cohort. **(B)** Gliomas and meningiomas. Solid line: gliomas; dotted line: meningiomas.

The 10-year survival rate was 49.4% (SE = 22.8%) for patients with secondary neoplasms (Figure 3A). The median follow-up period after the secondary-neoplasm diagnosis was 5.0 (range, 2.3–10.3) years for the seven patients who were alive at their latest follow-up. All patients with secondary meningiomas survived throughout the follow-up period. Three of the four patients with high-grade gliomas died as a result of the glioma at 1.9, 3.1, and 6.0 years, respectively, after the secondary-neoplasm diagnosis (Figure 3B), and the 3-year probability of survival among the four patients was 37.5% (SE = 28.6%).

## 4 Discussion

In our study, radiotherapy and CNS involvement in the primary cancer were significant risk factors for secondary neoplasms. However, we did not detect a significant relationship between female sex, younger age at the primary-cancer diagnosis, initial characteristics of the primary cancer, radiation dose, or allo-HCT and the cumulative incidence of secondary neoplasms, in contrast with previous studies (8, 10, 11, 13, 15–17). The 10- and 20-year cumulative incidences of secondary neoplasms were 2.9% (SE = 1.5%) and 5.5% (SE = 2.3%), respectively, among 188 long-term survivors of ALL and LBL. The risk of secondary neoplasms continued to increase over time and did not plateau, which is consistent with observations in previous reports (8–12).

Previous investigations demonstrated clear associations between secondary neoplasms and prior radiotherapy (9, 20–22). Our results are in agreement with those, considering that all patients who developed secondary neoplasms in our study had received prior CRT or TBI, whereas none of the patients who did not receive radiotherapy developed secondary neoplasms. Interestingly, the secondary neoplasms observed in our study were all CNS tumors, consisting of meningiomas (*n* = 6) and high-grade gliomas (*n* = 4), and no secondary neoplasms developed outside the irradiated field. The secondary neoplasms occurred 7.8–41.7 years from the primary-cancer diagnosis, and the median time to development of a secondary glioma was much shorter than that to development of a secondary meningioma (11.0 vs. 28.0 years, *p* = 0.047). The radiation-related increase in the incidence of glioma was apparent 5–10 years after radiotherapy, but largely disappeared after 15 years. In contrast, the cumulative incidence of meningioma continued to increase over time and did not plateau. This timing difference may be related to the age-specific background incidence rates and non-therapeutic exposures, such as tobacco, alcohol, and diet, related to meningiomas. The decrease in the relative risk of glioma over time is consistent with observations in a previous report of survivors of childhood cancer (23), in contrast with the results for atomic-bomb survivors (24) and patients irradiated for tinea capitis (25). Although the mechanisms underlying the early onset of glioma are largely unknown, cancer-predisposing conditions may exist. A detailed family history of cancer predisposition could not be obtained in this study, and further genetic investigations could not easily be performed because of the retrospective nature of our study. In a previous study, radiation dose–response relationships were observed for both gliomas and meningiomas (23). For doses in excess of 30 Gy, the relative risks were of the order of 20 for glioma and 50–100 for meningioma. Possibly owing to the small number of cases, no radiation dose-response relationship was observed in our study. Of 27 patients undergoing allo-HCT, 19 underwent TBI as a part of conditioning regimen and 2 of whom developed secondary gliomas. Further prospective studies are warranted to address this issue.

In our study, CNS involvement in the primary cancer was also a risk factor for the development of secondary CNS neoplasms. The cumulative incidence of secondary neoplasms at 20 years in patients with CNS involvement is 25% (SE = 21.7%). The large SE reflects the small sample size, nevertheless, this incidence is noteworthy. Patients with CNS involvement typically receive a higher radiation dose than those without. However, in our study, the irradiation doses for the two patients with CNS involvement who developed secondary CNS neoplasms were 12 and 18 Gy, respectively, and no difference in irradiation dose was observed compared with those with CNS involvement who did not develop secondary CNS neoplasms. Alteration of the microenvironment caused by blast-cell infiltration into the CNS may alter factors such as cytokines, nutrients, and oxygen supplied by the microenvironment, making it suitable for the development of secondary CNS neoplasms.

In our cohort, a relatively large number of patients, 63 of 188 (33.5%), underwent radiotherapy. At Mie University Hospital, prophylactic cranial irradiation was performed for high-risk patients with ALL and LBL until 2000, and prophylactic cranial irradiation was discontinued for primary ALL and LBL in 2001. Of the 63 patients treated with radiation therapy, 41 were treated before 2000, including 3 patients who received TBI as part of the allo-HCT conditioning regimen and 38 patients who received either prophylactic or therapeutic radiation. Twenty-two patients received radiotherapy after 2001, 16 for TBI, 3 for CNS involvement, and 3 for prophylactic cranial irradiation for relapsed ALL. Prophylactic cranial irradiation for relapsed cases was discontinued in 2015.

Several chemotherapeutic agents, especially alkylating agents and topoisomerase-II inhibitors, have been hypothesized to increase the risk of secondary neoplasms (12). Solid tumors are reportedly associated with cyclophosphamide exposure, and myeloid malignancy is reportedly associated with topoisomerase II inhibitors and higher starting doses of methotrexate and mercaptopurine (12). Cyclophosphamide was also associated with a threefold increased risk of secondary neoplasms in patients with ALL (14). Moreover, in another study, 11q23/MLL rearrangements were observed in one third of patients with treatment-related myeloid neoplasia with an aberrant karyotype and previous exposure to epipodophyllotoxins, which are topoisomerase II inhibitors (12). Therefore, we analyzed the relationship between chemotherapeutic agents (in addition to radiotherapy) and the incidence of secondary neoplasms. However, we did not observe a significant relationship between the cumulative dose of chemotherapeutic agents, such as alkylating agents, topoisomerase-II inhibitors (VP-16), and anthracyclines, and the incidence of secondary neoplasms.

At least one annual follow-up visit is offered to long-term childhood cancer survivors at our hospital. The long-term follow-up rate was relatively high in our cohort (approximately 80%), and those with secondary neoplasms were all treated and followed up at our hospital. This provided an accurate estimation of the risk of secondary neoplasms and enabled a detailed evaluation of the effect of treatment on secondary neoplasms. However, our results should be interpreted in the context of several limitations. First, because the data were based on a single center, the number of patients who developed secondary neoplasms was small, and the generalizability of the results are limited. Second, although the follow-up rate was relatively high, the number of patients with secondary neoplasms might have been underestimated and the result might have been skewed because of loss to follow-up and death due to any cause, including unreported secondary neoplasms. The information on how many patients were excluded from the study owing to a loss to follow up/death is important. However, the list of patients treated for ALL or LBL prior to 2000 has been lost. The 188 patients who were treated and still being followed up at Mie University Hospital were eligible for this study. Although the number of patients treated from 1977 to 2022 at Mie University Hospital is higher than 188, the exact number is unfortunately unknown. Third, radiation techniques have progressed since the 1970s, with the development of stereotactic radiotherapy, intensity-modulated radiotherapy, and particle-beam therapy. Therefore, discussing the radiotherapy-related risk of secondary neoplasms over the long study period is difficult.

In conclusion, our analysis confirmed radiotherapy and CNS involvement in the primary cancer as risk factors for secondary neoplasms. The risk of secondary neoplasms after successful treatment of childhood ALL and LBL was similar with that in previous studies. The development of secondary glioma plateaued by 15 years after irradiation, whereas the incidence of secondary meningioma increased after 10 years. As secondary gliomas are associated with poor outcomes, physicians should take great pains to minimize their risk to improve long-term survival and quality of life.

## Data Availability

The raw data supporting the conclusions of this article will be made available by the authors, without undue reservation.
